# Good Health-Related Quality of Life in Older Patients One Year after mTBI despite Incomplete Recovery: An Indication of the Disability Paradox?

**DOI:** 10.3390/jcm13092655

**Published:** 2024-05-01

**Authors:** Sophie M. Coffeng, Amaal Eman Abdulle, Harm J. van der Horn, Myrthe E. de Koning, Jan C. ter Maaten, Jacoba M. Spikman, Joukje van der Naalt

**Affiliations:** 1 Department of Emergency Medicine, University of Groningen, University Medical Center Groningen, 9700 RB Groningen, The Netherlands; 2 Department of Internal Medicine, University of Groningen, University Medical Center Groningen, 9700 RB Groningen, The Netherlands; a.eman.abdulle@umcg.nl (A.E.A.); j.c.ter.maaten@umcg.nl (J.C.t.M.); 3 Department of Neurology, University of Groningen, University Medical Center Groningen, 9700 RB Groningen, The Netherlands; h.j.van.der.horn@umcg.nl (H.J.v.d.H.); j.van.der.naalt@umcg.nl (J.v.d.N.); 4 Department of Neurology, Hospital Medisch Spectrum Twente, 7512 KZ Enschede, The Netherlands; me.dekoning@mst.nl; 5 Department of Clinical Neuropsychology, University of Groningen, University Medical Center Groningen, 9700 RB Groningen, The Netherlands; j.m.spikman@umcg.nl

**Keywords:** mild traumatic brain injury, aged, older, outcome assessment, health-related quality of life

## Abstract

**Background**: Older adults (OAs) with mild traumatic brain injury (OA-mTBI) are a growing population, but studies on long-term outcomes and quality of life are scarce. Our aim was to determine the health-related quality of life (HRQoL) in OA-mTBI one year after injury and to assess the early predictors of HRQoL. **Methods**: Data from a prospective follow-up study of 164 older (≥60 years) and 289 younger mTBI patients (<60 years) admitted to the emergency department were analyzed. Post-traumatic complaints, emotional distress and coping were evaluated 2 weeks post-injury using standardized questionnaires. At 12 months post-injury, HRQoL and functional recovery were determined with the abbreviated version of the World Health Organization Quality of Life scale and Glasgow Outcome Scale Extended (GOSE), respectively. **Results**: One year post-injury, 80% (n = 131) of the OA-mTBI rated their HRQoL as “good” or “very good”, which was comparable to younger patients (79% (n = 226), *p* = 0.72). Incomplete recovery (GOSE <8) was present in 43% (n = 69) of OA-mTBI, with 67% (n = 46) reporting good HRQoL. Two weeks post-injury, fewer OA-mTBI had (≥2) post-traumatic complaints compared to younger patients (68% vs. 80%, *p* = 0.01). In the multivariable analyses, only depression-related symptoms (OR = 1.20 for each symptom, 95% CI = 1.01–1.34, *p* < 0.01) were predictors of poor HRQoL in OA-mTBI. **Conclusions**: Similar to younger patients, most OA-mTBI rated their HRQoL as good at one year after injury, although a considerable proportion showed incomplete recovery according to the GOSE, suggesting a disability paradox. Depression-related symptoms emerged as a significant predictor for poor HRQoL and can be identified as an early target for treatment after mTBI.

## 1. Introduction

Mild traumatic brain injury (mTBI), accounting for 80–90% of all traumatic brain injuries, is a worldwide problem and a major cause of disability leading to substantial medical costs [1,2,3]. Over the last few decades, there has been a significant shift in the epidemiology of TBI, especially in high-income countries, with nearly doubling of the median age of TBI patients since the 1980s [1]. This shift is accompanied by changes in trauma mechanisms, with an increase in falls mostly occurring in this aging population [4,5,6]. Fall accidents in older patients are linked to age-associated physical factors, like balance disturbance, muscle weakness and the use of multiple medications [7,8,9]. Although falls from standing height constitute low-energy injury mechanisms, older patients show higher mortality and decreased rates of functional and cognitive recovery after sustaining a TBI compared to the younger population [10,11]. This is specifically related to an increased frequency of intracranial lesions due to the use of anticoagulants and the various comorbidities in older patients [10,12,13,14,15]. Furthermore, senescence as well as pre-existing conditions can contribute to extended hospital admission, hindering the recovery process and challenging physical and psychological recovery capacity [16].

Although there is increasing evidence of reduced functional and cognitive recovery in older patients with mTBI, studies on quality of life in this patient category are scarce. Health-related quality of life (HRQoL) is often described as a multi-dimensional concept that integrates the effects of an injury or medical condition on the quality of life of a patient [17]. HRQoL takes into account the subjective perspective of functional impairment, contrary to the Glasgow Outcome Scale-Extended (GOSE), the most widely used measure to assess functional outcomes after TBI [18]. Particularly in older individuals, who often experience pre-existing functional impairments, assessing functional outcomes is challenging. Previous research also showed associations between HRQoL and persistent post-traumatic complaints and emotional distress after TBI, but such studies in the older population are lacking [19,20,21]. It has been demonstrated that older patients have higher rates of depression relative to younger patients, which may also influence their ability to recover after mTBI [22].

The assessment of HRQoL and factors associated with HRQoL could provide valuable information regarding the recovery pattern of older patients with mTBI compared to traditional outcome measures. The aim of this study is therefore to determine the HRQoL of older mTBI patients and compare this to younger patients aged <60 years and to a general Dutch population cohort. In addition, we aim to assess if clinical characteristics, early post-traumatic complaints and indices of emotional distress are associated with HRQoL in older patients.

## 2. Materials and Methods

### 2.1. Participants

This study is part of a large observational cohort study on mTBI (the UPFRONT study) that recruited patients from 2013 to 2015 at the Emergency Department (ED) of three Dutch level-one trauma centers [23]. On arrival at the hospital, patients were recruited by the attending physician at the ED or at the ward after hospital admission. MTBI was defined by a Glasgow Coma Score (GCS) of 13–15, loss of consciousness (LOC) for ≤30 min and/or a duration of post-traumatic amnesia (PTA) of <24 h. The exclusion criteria were as follows: age <16 years at moment of injury, time since trauma > 24 h, addiction to drugs or alcohol, treatment for psychiatric disease, inability to follow-up (like a language barrier or no permanent home address). Patients without information about HRQoL were excluded. Patients were divided in two age groups: <60 years (younger) and ≥60 years (older). The decision to set the cut-off at 60 years aligns with the age CT-scanning criteria [24,25] and with a recent study indicating that individuals aged 60 and above face a higher risk of intracranial hemorrhage and adverse outcomes within 30 days after injury [26]. Data were collected from medical records and self-reports on demographic characteristics (sex, education level, medical history) and injury-related characteristics (mechanism of trauma, Computed Tomography (CT) characteristics, Injury Severity Scale). Comorbid disease was defined as the presence of one or more of the following diseases: cerebral vascular accidents, cardiovascular diseases, diabetes, hypertension, asthma, epilepsy and malignancy. The presence of pre-injury physical complaints was documented using the Symptom Checklist (SCL-90 revised) [27]. Pre-injury mental health problems were defined as psychiatric or psychological symptoms necessitating treatment by a psychiatrist or psychologist (either admission and/or psychotropic medication). 

With a 7-point scale, the patients’ Dutch education level was defined by years of education (YoE) [23]. For analysis, we dichotomized education as a low (finished secondary school (10–11 YoE or less)) or a high (finished secondary school (12–16 YoE) or university degree) educational level. This study was approved by the UMCG Medical Ethical Committee. All patients gave written informed consent. 

### 2.2. Clinical Measures 

For the current study, data from questionnaires (by mail or web-based) assessed at 2 weeks and 12 months post-injury were used. 

#### 2.2.1. Data Obtained at Two Weeks 

Post-traumatic complaints: The Head Injury Symptom Checklist (HISC) [28] assessed the 21 most common post-traumatic complaints, rating pre-injury and current complaints with sum scores ranging from 0 to 42 (0 = never, 1 = sometimes and 2 = often). Each symptom was corrected for the presence of the pre-injury symptom level by subtracting the pre-injury score from the score two weeks post-injury.

Emotional distress: The Hospital Anxiety and Depression Scale (HADS) [29] comprises fourteen items with seven items on depression and seven items on anxiety, with sum scores ranging from 0 to 21. A cut-off score of ≥8 indicates clinical anxiety (HADS-A) and clinical depression (HADS-D). 

Post-traumatic stress: The Impact of Event Scale (IES) [30] is a 15-item questionnaire with a 5-point Likert scale (sum scores 0–75). A cut-off score of ≥ 19 is indicative of post-traumatic stress disorder. 

Coping: The Utrecht Coping List (UCL) [31] is a 47-item questionnaire with seven subscales representing different coping styles. The scores range from low (1) to very high (5) use of specific coping styles. For the current study, the three most commonly reported scales were used (UCL-active, UCL-passive and UCL-avoidant), which is in line with other studies [23,31]. A cut-off score ≥ 4 was indicative of high use of a specific coping style. 

#### 2.2.2. Data Obtained at 12 Months

Health-related quality of life: HRQoL was measured with the Dutch version of the abbreviated version of the World Health Organization Quality of Life scale (WHOQOL-BREF) [32,33]. WHOQOL-BREF has two parts; the first contains two general items on the perception of quality of life and on the perception of general health, and the second includes 24 items assessing four domains of HRQoL: physical health (7 items), psychological health (6 items), social relationships (3 items) and environment (8 items). The two general questions are ‘How would you rate your quality of life?’ and ‘How satisfied are you with your health?’. Each item is rated on a 5-point scale (1 = very poor, 5 = very good). Domain scores are calculated by multiplying the mean score within each domain by four resulting in domain scores (ranging from 4 to 20). The higher the score in a domain, the better the HRQoL in that specific area. Since a healthy control group was not included in the UPFRONT study, data were compared with data of 271 persons aged ≥60 years and 354 persons ages 18–59 years of the general Dutch population based on the WHOQOL-BREF [34]. For univariate and multivariable analyses, the general perceived HRQoL scores were dichotomized into good (scores of 4 (good) and 5 (very good)) and poor (scores of 1 (poor) to 3 (neither poor nor good)). 

Functional outcome: The GOSE [18] determined functional outcomes using an eight-point scale (8 = complete recovery, 7 = incomplete recovery, 6 = upper moderate disability, 5 = lower moderate disability, 4 = upper severe disability, 3 = lower severe disability, 2 = vegetative state, 1 = death). Outcomes were dichotomized as complete (GOSE = 8) or incomplete recovery (GOSE < 8).

### 2.3. Statistical Analysis

Analyses were performed with Statistical Package for the Social Sciences version 28.0 (SPSS). Demographic and trauma characteristics are described using mean and standard deviation (SD) or median and interquartile range (IQR) for continuous variables and frequencies and percentages for categorical variables. Differences between younger and older patients were tested with Student’s *t* test or a nonparametric Mann–Whitney U-test. Nominal statistics were obtained with the Pearson χ^2^ test. A two-tailed probability <0.05 was considered significant. Univariate logistic regression was performed with dichotomized perceived HRQoL as the dependent variable for the older mTBI group. The following variables for regression analyses were chosen based on previous research: patient and trauma characteristics (sex, educational level, GCS score, pre-injury mental health, comorbid diseases, CT abnormalities, living situation) and assessments two weeks post-injury (coping mechanism, number of post-traumatic complaints, emotional distress) [15,23,35,36]. For univariate and multivariable analyses, the continuous scores of the following variables were used: post-traumatic complaints, emotional distress and post-traumatic stress. To minimize potential bias, missing data were assumed to be missing at random. Therefore, missing data of general characteristics were imputed based on all available clinical risk factors using multiple imputation (n = 5) with the ‘multivariable imputation by chained equations’.

## 3. Results

### 3.1. Patient Characteristics

**Figure 1:**
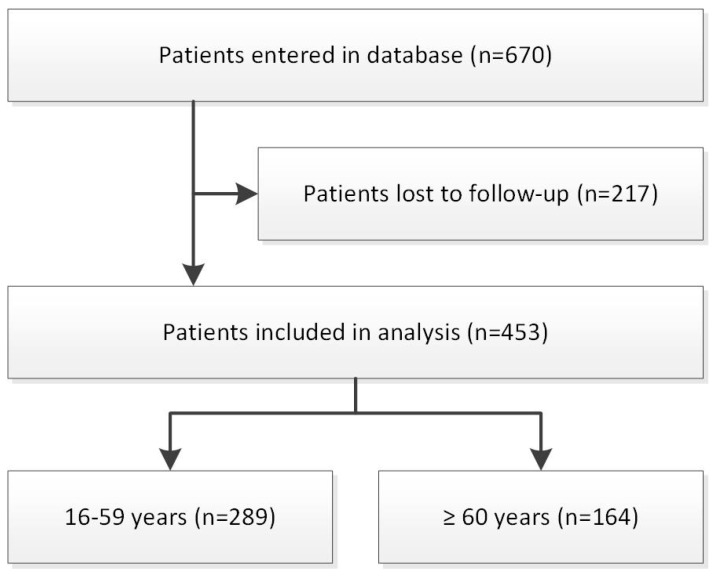
Flowchart.

In total, data from 453 patients (older patients, n = 164; younger patients, n = 289) were obtained from completed questionnaires at 12 months post-injury (Figure 1). The included patients showed no significant differences compared to patients who were lost to follow-up (n = 217) regarding age (50 ± 18 SD versus (vs.) 48 ± 19 years, *p* = 0.192), sex (60% vs. 61% male, *p* = 0.762) and injury severity score (7 ± 6 vs. 8 ± 5, *p* = 0.201). Table 1 shows the demographic and trauma characteristics of older and younger patients, and Supplementary Table S1 shows the imputed demographic and trauma characteristics of elderly patients.

### 3.2. Post-traumatic Complaints, Emotional Distress and Coping at 2 Weeks Post-Injury

At two weeks post-injury, most patients (333, 76%) had two or more post-traumatic complaints, including 69% (n = 108) of the older and 80% (n = 225) of the younger mTBI groups (*p* = 0.01).

Regarding emotional distress, 15% of the older patients scored above the cut-off for anxiety (HADS-A ≥ 8) and 15% above the cut-off score for depression (HADS-D ≥ 8) two weeks post-injury. No difference was present between older and the younger patients in the frequency of depression (15% vs. 16%, *p* = 0.74) or anxiety (15% vs. 14%, *p* = 0.74). In total, 38% of all patients suffered from post-traumatic stress (IES), without significant differences between older and younger patients (43% vs. 34%, *p* = 0.08).

Concerning coping styles, the avoidant coping style was the most frequently used coping style in both age groups (28% older patients, 34% younger patients, *p* = 0.170). Older patients less frequently showed a high use of passive coping compared to younger patients (19% vs. 28%, *p* = 0.04). Regarding active coping, no differences were found between older and younger patients (26% vs. 31%, *p* = 0.28).

### 3.3. Health-Related Quality of Life and Functional Recovery

**Figure 2:**
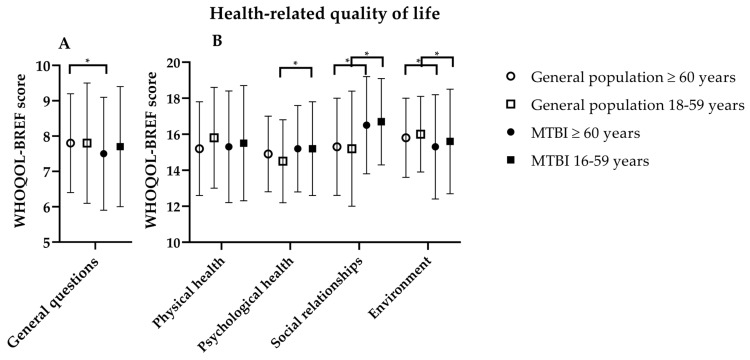
Health-related quality of life domains for older and younger patients one year post-mTBI and for the general Dutch population. Legend: (**A**) WHOQOL-BREF cumulative scores of the 2 general questions: 1. general perception of life, 2. satisfaction with health. (**B**) WHOQOL-BREF scores of the 4 domains. * significant difference between groups.

The majority of the mTBI patients rated their HRQoL 12 months after injury as “good” or “very good” (Table 2), without a difference between the older (80%) and younger patients (78%; *p* = 0.73). Considering their perception of general health, which is the second question of the WHOQOL-BREF, older patients less frequently reported “good” or “very good” responses compared to younger patients (56% vs. 66%, *p* = 0.03). The domain scores of the HRQoL showed no significant differences between older and younger mTBI patients (Figure 2).

When compared to the general Dutch population (aged ≥60 years), older mTBI patients scored lower on the accumulated score of the two general health questions (7.5 ± 1.6 vs. 7.8 ± 1.4, *p* = 0.04) and in the environment subdomain (15.3 ± 2.9 vs. 15.8 ± 2.2, *p* < 0.01), but relatively higher in the subdomain of social relationships (16.5 ± 2.7 vs. 15.3 ± 2.7, *p* = 0.04). With respect to younger mTBI patients, they scored higher than the general Dutch population (aged <60 years) in the psychological (15.2 ± 2.6 vs. 14.5 ± 2.3, *p* < 0.01) and in the social relationship subdomains (16.7 ± 2.4 vs. 15.2 ± 3.2, *p* < 0.01), but lower in the environmental subdomain (15.6 ± 2.9 vs. 16.0 ± 2.1, *p* = 0.04) (Figure 2).

Twelve months post-injury, 40% of the total cohort (n = 439) showed incomplete recovery (GOSE < 8). The percentages for older and younger patients were comparable (43% vs. 39%, *p* = 0.44). Of the older patients with incomplete recovery, 67% (n = 46) experienced good HRQoL at one year compared to 53% (n = 57) in the younger group (*p* = 0.07) (Table 3).

### 3.4. Predictors of One-Year Post-Injury HRQoL for Older and Younger Age Groups

Table 4 shows the univariate and multivariable analysis results for one-year HRQoL in older patients. Dizziness was not associated with HRQoL in the univariate analysis (odds ratio (OR) = 1.06 (95% confidence interval (CI) = 0.47–2.41) *p* = 0.887). Multivariable analysis in this group showed that only higher depression scores (OR = 1.20 for each depressive symptom on the questionnaire, 95% CI = 1.01–1.34, *p* < 0.01) were associated with a poor HRQoL one year post-injury. Supplementary Table S2 shows the same analysis for the younger group. In the younger group, poor HRQoL was associated with pre-injury mental health (OR = 6.64, 95% CI = 2.21–20.0, *p* < 0.01), more anxiety symptoms (OR = 1.13, 95% CI = 1.02–1.26, *p* = 0.02) and more depression symptoms (OR = 1.20, 95% CI = 1.09–1.32, *p* < 0.01).

## 4. Discussion

The current study evaluated health-related quality of life and its early predictors in older patients with mTBI at one year post-injury. The majority of older mTBI patients reported good HRQoL, which was similar to younger patients. Particularly in older patients, the presence of early depression-related symptoms two weeks after sustaining mTBI was associated with poor HRQoL, which occurred in 20% of patients. This finding offers the possibility to identify patients at risk for poor HRQoL at an early stage after injury.

The current study showed that most older patients reported good HRQoL one year after sustaining a mTBI. In fact, there were no significant differences compared to the younger mTBI patients regarding the overall perception of HRQoL nor in the distinct HRQoL domains of physical health, psychological health, social relationships and the environment. Earlier TBI studies have reported contradicting results with respect to the influence of age on HRQoL. One study showed that young adults (aged 18–35 years) were especially at risk of a poorer quality of life six months after sustaining mTBI [37]. A recent study reported lower HRQoL up to six months post-injury in older mTBI patients compared to non-injured older adults [38]. This last study seems to contradict our current results, which could be due to the fact that their study involved a relatively small population with 92% of the elderly patients reporting their HRQoL to be normal. Older age has also been found to be a strong independent predictor of poor HRQoL in severe TBI [36,39]. It is conceivable that older patients with severe TBI lack the resilience to combat symptoms, resulting in poor HRQoL. Our study revealed that this does not apply to older patients with mTBI.

Because of the overall high scores regarding HRQoL, we also evaluated the scores of the subdomains. The subdomain scores were comparable between age groups, although older mTBI patients scored significantly lower in the environmental domain compared to older adults in the general Dutch population. The same applies to younger patients. The environmental domain contains questions about security, financial support, recreation and the availability of medical services and transportation. A possible explanation for the lower scores in this domain could be that elderly patients have less access to these environmental aspects due to their injury. The scores in the other subdomains were similar (physical health and psychological health) or even higher (social relationships) in mTBI patients relative to older adults of the general Dutch population. This was unexpected, as we assumed beforehand that mTBI would affect HRQoL one year post-injury, especially in older patients. A possible explanation for this finding might be that behavioral adaptation is more prevalent in older patients who already experience more physical complaints, with less focus on the resolution of (new) post-traumatic complaints and returning to their previous functional status. In addition, we hypothesize that older people, due to aging and comorbidities, had already changed their needs regarding their achievements in everyday life, and therefore might better be able to successfully adapt to new functional decline. In line with this reasoning, our results showed that older mTBI patients perceived their health status as relatively lower compared to the general older Dutch population, but nonetheless experience good HRQoL.

The aforementioned adaptive behavior might also be an indication of coping style, which is another factor that should be considered when assessing HRQoL. Maladaptive coping, especially a passive coping style, is associated with incomplete recovery after mTBI [23]. Interestingly, this was the only coping style which was used significantly less frequently in the older group compared to their younger counterparts. By using a passive coping style less frequently, older patients might adapt more easily after experiencing mTBI. Conversely, the increased use of a passive coping style in the younger patients of our cohort might explain the association of other factors (like pre-injury mental health and emotional distress) with HRQoL, suggesting another psychological coping profile in this stage of life.

Interestingly, the perception of HRQoL after mTBI does not exactly seem to match with the status of functional recovery measured by the GOSE in our cohort of older mTBI patients. More than half of the older patients with incomplete recovery reported good HRQoL, referred to as the disability paradox. A recent study reported that especially mTBI patients with severe disability reported high HRQoL [40]. This discordance between disability and HRQoL was postulated to be associated with perceived social support. Although we did not evaluate social support in our cohort, the increased scores in the social relationship domain compared to the general Dutch population might be considered indirect evidence for our ideas. It is known that the GOSE is a clinically relevant instrument to monitor functional recovery, but fails to capture the subjective perspective of patients related to psychological and environmental aspects of quality of life [41]. In addition, the needs of patients change throughout their lifetime, and the GOSE questionnaire does not take into account this change in needs. The HRQoL, which includes more subjective questions, might therefore be of added value to the GOSE in assessing post-injury outcomes following mTBI.

Despite the finding of an overall high level of HRQoL, one in five older mTBI patients in our cohort still experienced poor HRQoL one year post-injury. Therefore, it is important to find risk factors that identify these patients at an early stage, creating an opportunity for tailored treatment to increase their long-term satisfaction in life. The only predictor of poor HRQoL in older mTBI patients in our study was the presence of early depression-related symptoms two weeks post-injury. Previous research has shown a significant association of depression with incomplete recovery and HRQoL after mTBI irrespective of age [23,42]. Due to their negative perception and interpretation of their sustained injury, patients with depression are at risk of experiencing more post-traumatic complaints, which affects outcomes negatively [42,43,44]. Depression can also impair functioning in other areas than health, such as social functioning, with a subsequent impact on HRQoL [45]. It is also likely that depression hampers the employment of adaptive behavior in older patients. Remarkably, other factors, like post-traumatic complaints, were not found to be associated with a poor HRQoL in our population of older patients. An explanation for this observation might be that for older patients it is more difficult to distinguish between post-traumatic and pre-existing complaints that are associated with aging. One example of a common post-traumatic complaint which is difficult to distinguish from being present pre- and postinjury is dizziness. Dizziness is a prevalent condition among older patients, frequently manifesting as chronic multi-canalicular benign paroxysmal positional vertigo, and is associated with previous trauma [46,47]. We examined dizziness separately as part of the HISC questionnaire and found this not to be associated with HRQoL. However, a specific dizziness questionnaire was not used, like the Dizziness Handicap Inventory, and therefore, a proper assessment of vertigo is lacking [48]. This could be an interesting subject for future research.

To conclude, the identification of patients at risk for a poor HRQoL should preferably be carried out at an early stage, as depression is frequently undertreated in TBI patients, with the potential consequence of poor HRQoL one year post-injury [49]. Screening at an early stage of post-injury, either by telephonic consultation or at outpatient clinics by health care providers, could provide information on the presence of risk factors for the development of depression after mTBI.

Certain limitations of this study should be addressed. First, a substantial number of patients were lost to follow-up, which could have biased our results towards worse outcomes [50,51]. However, despite these drop-out rates, no significant differences were present between dropouts and patients with complete follow-ups regarding baseline characteristics. The number of drop-outs in our study is also comparable with other longitudinal mTBI follow-up studies, facilitating comparison of results [52,53]. Second, information on specific needs in daily life or the presence of frailty that could interact with HRQoL were not available. This information might have shed more light on the difference between age groups regarding long-term HRQoL. Further research is needed on the contributing value of these environmental variables. Third, some variables were dichotomized for statistical analysis, which could theoretically have led to the loss of information. Fourth, since this study was part of the UPFRONT study, a separate sample size calculation was not conducted for the HRQoL questionnaire and its subdomains. However, when considering other studies examining older adults and HRQoL, our group is comparable or even larger [38,54,55]. Therefore, we believe that our sample size was sufficient to provide a reliable assessment of HRQoL, although we might have lacked power for detecting differences at the subdomain level. Lastly, the assessment of HRQoL in our cohort of older adults was conducted around 2015/2016. Due to technological advancements in recent years, theoretically, the quality of life of older adults may have improved. To our knowledge, there are no recent studies available, conducted in the Netherlands or Europe, that have examined HRQoL using the WHOQOL-BREF in older patients to confirm this hypothesis. The only relevant finding was a measurement by the Dutch Ministry of Health, Welfare and Sport, indicating that the overall quality of life among older patients was in the range of 65–69% between 2019 and 2021, which was not measured by the WHOQOL-BREF [56]. Additionally, HRQoL in our cohort was high, and we expect that any potential improvement would not have altered our findings that older adults generally rate their HRQoL as good after MTBI. Despite these limitations, it must be acknowledged that this large cohort study is one of the first studies to investigate HRQoL and its predictors in older patients with mTBI one year post-injury.

## 5. Conclusions

The current study showed that the majority of older patients with mTBI reported a high level of health-related quality of life one year post-injury. In half of the patients, this was accompanied by incomplete recovery, a so-called disability paradox. Still, one in five patients reported poor HRQoL, with early depression-related symptoms emerging as an important determinant of the perceived lower HRQoL. The identification of this risk factor at early-stage post-injury might facilitate adequate support and treatment to improve HRQoL in the long term.

## Figures and Tables

**Table 1:** Demographics and trauma characteristics of the mTBI cohort.

	Total (n = 453)	Older Patients (n = 164)	Younger Patients (n = 289)	*p*-Value *	Missing
Age, mean ± sd	49.2 ± 18.2	68.6 ± 7.0	39.2 ± 13.4	<0.001	0
Sex (male), n (%)	270 (59.6)	98 (59.8)	172 (59.5)	0.96	0
Comorbidities, n (%)	155 (34.2)	105 (64.0)	50 (17.3)	<0.001	0
GCS = 15, n (%)	297 (65.6)	116 (70.7)	181 (62.6)	0.07	0
Hospital admission, n (%)	283 (62.5)	113 (68.9)	170 (58.8)	0.03	0
Mechanism of injury, n (%)				0.35	0
Collision	108 (23.8)	31 (18.9)	77 (26.6)		
Fall	309 (68.2)	124 (75.6)	185 (64.0)		
Other cause	36 (7.9)	9 (5.5)	27 (9.3)		
Discharged to home, n (%)	434 (95.8)	157 (95.7)	277 (95.8)	0.95	0
ISS, mean ± sd	7.3 ± 5.2	7.7 ± 5.2	7.2 ± 5.2	0.38	46
CT abnormalities, n (%)	78 (17.2)	33 (20.1)	45 (15.6)	0.22	0
Pre-injury physical ^†^, n (%)	112 (24.7)	56 (34.1)	56 (19.4)	<0.001	15
Pre-injury mental health ^†^, n (%)	33 (7.3)	11 (6.7)	22 (7.6)	0.80	17
Educational level high, n (%)	220 (50.5)	64 (42.1)	156 (54.9)	0.01	17
Living alone, n (%)	86 (19.5)	29 (18.5)	57 (20.1)	0.60	12

Legend: Results are represented as numbers with percentages if not indicated otherwise. Older patients: aged ≥60 years; younger patients: aged <60 years; GCS: Glasgow Coma Scale; ISS: Injury Severity Score; CT: Computed Tomography; sd: standard deviation; * *p*-value of the difference between the older and younger mTBI age groups; ^†^ pre-injury mental or physical health complaints.

**Table 2:** General perception of one-year post-injury health-related quality of life and satisfaction with overall health after mTBI.

Answer Scale	Perception of HRQoL			Satisfaction with Health		
	Older Patients (n = 164)	Younger Patients (n = 288 *)	*p*-Value	Older Patients (n = 164)	Younger Patients (n = 288 *)	*p*-Value
Poor (1–3)	33 (20.1)	62 (21.5)	0.724	72 (43.9)	97 (33.7)	0.031
Good (4–5)	131 (79.9)	226 (78.5)		92 (56.1)	191 (66.3)	

Legend: Results are represented as numbers with percentages. Older patients: aged ≥60 years. Younger patients: aged <60 years. HRQoL: health-related quality of life. mTBI: mild traumatic brain injury. * one missing value.

**Table 3:** Relationship between health-related quality of life and recovery 12 months post-injury.

Health-Related Quality of Life		
	Poor	Good
**Total group (N = 439)**		
*Incomplete recovery*	74 (41.8%)	103 (58.2%)
*Complete recovery*	19 (7.3%)	243 (92.7%)
**Older patients (N = 161)**		
*Incomplete recovery*	23 (33.3%)	46 (66.7%)
*Complete recovery*	10 (10.9%)	82 (89.1%)
**Younger patients (N = 278)**		
*Incomplete recovery*	51 (47.2%)	57 (52.8%)
*Complete recovery*	9 (5.3%)	161 (94.7%)

Legend: incomplete recovery = Glasgow outcome scale extended <8; complete recovery = Glasgow outcome scale extended = 8; older patients: aged ≥60 years; younger patients: aged <60 years.

**Table 4:** Univariate and multivariable analysis of the predictors of one-year post-injury HRQoL for older patients (≥60 years).

		Poor One-Year Post-Injury HRQoL Perception of Older Patients (N = 164)					
		Univariate			Multivariable		
	Coding	OR	95% CI	*p*-Value	OR	95% CI	*p*-Value
*Baseline Data*							
Sex	Male (0)–Female (1)	0.82	0.37–1.80	0.61	NS	NS	NS
Education level	Low (0)–High (1)	0.67	0.30–1.49	0.33	NS	NS	NS
Living alone	No (0)–Yes (1)	2.56	1.06–6.23	0.04 *	NS	NS	NS
Pre-injury physical complaints	No (0)–Yes (1)	1.77	0.81–3.84	0.15	NS	NS	NS
Pre-injury mental health	No (0)–Yes (1)	0.88	0.18–4.26	0.87	NS	NS	NS
GCS score ED	<15 (0)–15 (1)	1.06	0.46–2.45	0.88	NS	NS	NS
CT abnormalities	No (0)–Yes (1)	0.86	0.32–2.82	0.76	NS	NS	NS
ISS score	0–39	0.94	0.85–1.04	0.20	NS	NS	NS
*Two weeks post-injury*							
Active coping style	No (0)–Yes (1)	0.52	0.18–1.46	0.21	NS	NS	NS
Passive coping style	No (0)–Yes (1)	1.08	0.40–2.93	0.89	NS	NS	NS
Avoidant coping style	No (0)–Yes (1)	0.59	0.22–1.57	0.29	NS	NS	NS
Post-traumatic complaints	0–25	1.17	1.07–1.29	0.01 *	NS	NS	NS
Anxiety scores	0–15	1.08	0.97–1.19	0.16	NS	NS	NS
Depression scores	0–18	1.21	1.09–1.35	<0.01 *	1.20	1.01–1.34	<0.01 *
Post-traumatic stress	0–73	1.01	0.98–1.03	0.64	NS	NS	NS

Legend: CT: Computed Tomography; GCS = Glasgow Coma Scale; HRQoL = health-related quality of life; NS = not significant; * significant *p*-value.

## Data Availability

Anonymized patient-level data are available upon reasonable request after contact with J.N.

## Supplementary Materials

The following supporting information can be downloaded at: https://www.mdpi.com/article/10.3390/jcm13092655/s1, Table S1: Demographics and trauma characteristics of the patient group after imputation. Table S2: Univariate and multivariable analysis of the predictors of one-year post-injury HRQoL for younger patients (<60 years).
