# Supplementary material for: Good Health-Related Quality of Life in Older Patients One Year after mTBI despite Incomplete Recovery: An Indication of the Disability Paradox?
Source: J Clin Med. 2024 May 1;13(9):2655. doi: 10.3390/jcm13092655 (PMC11084863; doi:10.3390/jcm13092655)
Supplement: Supplementary file 1 [file jcm-13-02655-s001.zip › jcm-2956753-supplementary.pdf]

Supplemental Table S1: Demographic and trauma characteristics of the patient group after imputation.

	**Total (n=453)**	**Older mTBI patients (n=164)**	**Younger mTBI patients (n=289)**	**p-value***
**Age, mean ±sd**	49.2 ± 18.2	68.6 ± 7.0	39.2 ± 13.4	**<0.001**
**Sex (Male), n(%)**	270 (59.6)	98 (59.8)	172 (59.5)	0.96
**Comorbidities, n(%)**	155 (34.2)	105 (64.0)	50 (17.3)	**<0.001**
**GCS=15, n(%)**	297 (65.6)	116 (70.7)	181 (62.6)	0.07
**Hospital admission, n(%)**	283 (62.5)	113 (68.9)	170 (58.8)	**0.03**
**Mechanism of injury, n(%)**				0.35
Collision	108 (23.8)	31 (18.9)	77 (26.6)	
Fall	309 (68.2)	124 (75.6)	185 (64.0)	
Other cause	36 (7.9)	9 (5.5)	27 (9.3)	
**Discharged to home, n(%)**	434 (95.8)	157 (95.7)	277 (95.8)	0.95
**ISS, mean ± sd**	7.3 ± 5.2	7.6 ± 4.9	7.2 ± 5.0	0.36
**CT-abnormalities, n(%)**	78 (17.2)	33 (20.1)	45 (15.6)	0.24
**Pre-injury physical†, n(%)**	114 (24.7)	57 (34.8)	57 (19.7)	**<0.001**
**Pre-injury mental health†, n(%)**	33 (7.3)	11 (6.7)	22 (7.6)	0.72
**Educational level high, n(%)**	227 (50.1)	67 (40.9)	160 (55.4)	**<0.01**
**Living alone, n(%)**	86 (19.0)	29 (17.7)	57 (19.7)	0.60

Legend: Results are represented as number with percentages if not indicated otherwise. Older patients: aged ≥ 60 years, Younger patients: aged < 60 years, GCS: Glasgow Coma Scale, ISS: Injury Severity Score, CT: Computed Tomography, sd: standard deviation, * p-value of the difference between age-groups, † Pre-injury mental or physical health complaints.

Supplemental Table S2: Univariate and multivariable analysis of the predictors of one year post-injury HRQoL for younger patients (< 60 years).

		Poor one year post-injury HRQoL perception of younger patients (N=289)					
		Univariate			Multivariable		
	**Coding**	**OR**	**95% CI**	**p-value**	**OR**	**95% CI**	**p-value**
Baseline data							
**Gender**	Male (0) – Female (1)	1.53	0.87-2.69	0.14	NS	NS	NS
**Education level**	Low (0) – High (1)	0.72	0.41-1.28	0.26	NS	NS	NS
**GCS score ED**	< 15 (0) – 15 (1)	1.53	0.87-2.71	0.14	NS	NS	NS
**Pre-injury physical complaints**	No (0) – Yes (1)	2.85	1.50-5.41	<0.01	NS	NS	NS
**Pre-injury mental health**	No (0) – Yes (1)	10.24	3.95-26.6	<0.01	6.64	2.21-20.0	<0.01
**CT-abnormalities**	No (0) – Yes (1)	2.07	1.03-4.15	0.04	NS	NS	NS
Two weeks post-injury							
**Active coping style**	No (0) – Yes (1)	0.76	0.40-1.54	0.40	NS	NS	NS
**Passive coping style**	No (0) – Yes (1)	3.30	1.80-6.06	<0.01	NS	NS	NS
**Avoidant coping style**	No (0) – Yes (1)	0.91	0.49-1.68	0.77	NS	NS	NS
**Posttraumatic complaints**	0-27	1.14	1.06-1.22	<0.01	NS	NS	NS
**Anxiety scores**	0-19	1.27	1.17-1.39	<0.01	1.13	1.02-1.26	0.02
**Depression scores**	0-19	1.29	1.19-1.40	<0.01	1.20	1.09-1.32	<0.01
**Posttraumatic stress**	0-61	1.05	1.03-1.07	<0.01	NS	NS	NS

Legend: CT: Computed Tomography, GCS = Glasgow Coma Scale, ISS = Injury Severity Score, HADS-A/D: Hospital Anxiety and Depression Scale- Anxiety or Depression, HRQoL = Health related quality of life.
